# Analysis of the Status and Trends of Chinese Clinical Practice Guideline Development Between 2010 and 2020: A Systematic Review

**DOI:** 10.3389/fmed.2021.758617

**Published:** 2021-11-02

**Authors:** Rong Zhang, Si-yu Yan, Yun-yun Wang, Qiao Huang, Xiang-ying Ren, Ran Tan, Yu-qing Deng, Lin-xia Su, Yong-bo Wang, Zheng-rong Zhao, Ying-hui Jin

**Affiliations:** ^1^Center for Evidence-Based and Translational Medicine, Zhongnan Hospital of Wuhan University, Wuhan, China; ^2^Department of Neurotumor Disease Diagnosis and Treatment Center, Taihe Hospital, Hubei University of Medicine, Shiyan, China; ^3^School of Health Sciences, Wuhan University, Wuhan, China; ^4^Department of Evidence-Based Medicine and Clinical Epidemiology, The Second Clinical College, Wuhan University, Wuhan, China; ^5^School of Nursing and Health, Henan University, Kaifeng, China; ^6^Department of Breast Surgery, General Surgery, Qilu Hospital of Shandong University, Jinan, China; ^7^Department of Urology, Central Hospital of Wuhan, Tongji Medical College, Huazhong University of Science and Technology, Wuhan, China; ^8^Department of Neurology, Qilu Hospital of Shandong University, Jinan, China; ^9^College of Acupuncture and Orthopedics, Hubei University of Chinese Medicine, Wuhan, China

**Keywords:** clinical practice guidelines, EB-CPGs, CB-CPGs, methodological quality, GRADE

## Abstract

**Objective:** This study aimed to systematically review the status and trends of Chinese clinical practice guidelines (CPGs) during the time period 2010–2020 and explore their methodological characteristics. Then, based on the strengths and weaknesses in development, offer several recommendations for the quality improvement which will serve as a reference for the users and developers of CPG.

**Introduction:** With the development of evidence-based medicine (EBM), the CPGs play an increasingly important role in healthcare decision-making both in China and worldwide.

**Inclusion criteria:** The CPGs that have been used to help the health professionals in the healthcare decision-making were included.

**Methodology:** The China National Knowledge Infrastructure (CNKI) and WanFang databases were searched from 2010 to 2020 for the studies describing the general and methodological characteristics of Chinese CPGs. Comparisons of the methodological characteristics between the groups were conducted using the chi-square test or Fisher's exact test. The M-K test was adopted to identify the monotonically increasing or decreasing trends of methodological characteristics over the timespan.

**Results:** A total of 2,654 CPGs fulfilled the inclusion criteria. The quantity and quality of the guidelines developed in China have improved over the time span. From 2010 to 2020,the guidelines had differing characteristics and covered a wide range of subjects. In total, 2,318(87.34%) guidelines focused on Western Medicine. Eight (0.30%) had been developed for patient versions of guidelines, 10(0.38%) were tentative guidelines, and 16(0.60%) were rapid advice guidelines. Medical specialty societies (including their branches) (71.1%) were the main guideline makers. The most addressed diseases were neoplasms (14.43%). The target population is mainly adults (84.97%). The methodological quality of consensus-based (CB)-CPGs was obviously lower than evidence-based (EB)-CPGs. Except for the item, “recommendations were based on evidence of systematic reviews,” there were statistical differences in all other methodological items between the EB-CPGS and CB-CPGS (*P* < 0.01). Higher methodological quality has been observed in EB-CPGs. All the data relating to the methodological characteristics indicated that higher methodological quality was present in the guidelines using GRADE (*P* < 0.01).

**Conclusion:** The quantity and quality of the guidelines developed in China have improved between 2010 and 2020. CB-CPGs have also paid attention to the methodology quality, but obviously, this is lower than that in the EB-CPGs.

## Introduction

The clinical practice guidelines (CPGs) have become increasingly prominent in clinical medicine and represent one of the most important tools for potentially improving clinical decision-making and patient outcomes (1, 2). They are statements that include the recommendations for the optimization of patient care and are informed by a systematic review of evidence and an assessment of the risks and befits of alternative options (1). As the most important guiding documents in the medical practice, the CPGs provide specific recommendations based on the available research and are useful in informing evidence-based practice. The CPGs have become a very popular tool for decision making in healthcare. In China, during the last 25 years as a way of transmission and development of using the best available evidence to direct clinical decision-making, the development of CPGs has received widespread attention in the academic fields and grown exponentially in recent years (3–5). A national cross-sectional survey in China reported that nearly all the participants considered guidelines to provide essential or basic guidance for healthcare delivery and 1,313 (77.1%) participants reported frequent or very frequent use of guidelines with 61.8% of participants using the Chinese guidelines (6).

However, the previous studies assessing some CPGs in China have indicated that improvements in the quality, transparency, and usability of the guidelines are required which included identifying, appraising, and synthesizing the evidence underpinning the guideline recommendations, and paying more attention in dealing with and reporting conflicts of interest (3–5, 7–12). The whole situation of CPGs development is unclear, especially in the recent decade including the extent to which CPGs in China currently utilize rigorous methods. This study aimed to describe the status and trends of Chinese CPGs during the time period 2010–2020 and explore their methodological characteristics. Then, based on the strengths and weaknesses in development, offer several recommendations for quality improvement which will serve as a reference for the users and developers of CPG.

## Methods

### Approach

We conducted a systematic review of studies that described the general and methodological characteristics of Chinese guidelines. Reporting of the methods and findings were guided by the Preferred Reporting Items for Systematic Reviews and Meta-Analyses (PRISMA) criteria.

### Research Question

(1) What are the general characteristics (e.g., number of publications, guideline classification, and theme)? (2) What are the methodological characteristics which included sources of evidence, criteria for grading the quality of evidence and strength of recommendations, developing recommendations, sources of guideline funding, and conflicts of interest? (3) What are the differences in the methodological characteristics between the evidence based (EB)-CPGs and consensus-based (CB)-CPGs? (4) What are the differences in the methodological characteristics between GRADE and Non-GRADE CPGs?

### Identifying Relevant CPGs

#### Search Strategy

The China National Knowledge Infrastructure (CNKI) and WANFANG were searched for all the guidelines in China. The keywords for the searches included Chinese words for terms, such as “guidelines,” “practice guideline,” “clinical guideline,” “clinical practice guideline,” “consensus,” “expert consensus,” “expert consensus statements,” “professional consensus,” and “recommendation.” We searched for these terms in the title fields. Considering the time lag for CPGs being included in a database, the time scope of the search was from January 2010 to June 2021.

#### Eligibility Criteria

We identified the EB-CPGs and CB-CPGs published in China available in the Chinese electronic databases including China National Knowledge Infrastructure (CNKI) and WANFANG during the time period of January 2010–December 2020. The inclusion criteria were: the articles were considered as guidelines if they met the definition of a guideline as proposed by the Institute of Medicine (IOM) (1, 13). We classified the guidelines into two types based on their title definition of CPGs (EB-CPGs and CB-CPGs). Usually, when evidence is only of low quality or very low quality, the guideline panels label them as consensus statements and expert opinions. In this research, we describe both the consensus statements and expert opinions as CB-CPGs.

If several versions of one guideline existed, only the version that included the greatest detail on the guideline development methodology was assessed. If a guideline had updates, the previous version and updated version both were assessed. If one guideline was published in several parts, we merged them into one complete guideline for the assessment. The systematic reviews, editorials, translations, compilations, adaptions, interpretations, and short summaries for the guideline were excluded.

#### Guidelines Selection

First, title and abstract screening was independently performed by the two reviewers (Yan S-y and Ren X-y). Only clearly irrelevant literature was excluded at this stage. Second, for all the potentially relevant guidelines, the publications were obtained and checked for final inclusion by the two reviewers (Wang Y-y and Ren X-y) independently. Disagreement was resolved through the discussion with a third author (Jin Y-h). Any discrepancies were discussed and resolved through the consensus.

### Data Extraction

The data were extracted using a double-extraction method from each eligible CPG and its corresponding **Appendices** by the two reviewers (Zhang R and Wang Y-y) who are familiar with evidence-based medicine (EBM) and guideline development methodology. Any disagreement was also resolved through the discussion with a third author (Jin Y-h). The variables which comprised the data extraction table included the general and methodological characteristics of the Chinese CPGs and were generated from those characteristics named in the AGREE instrument which were of interest to us. The following data were extracted to present the general characteristics: guideline title, journal name, year of publication, number of publications, update, development body (National Health Commission of the People's Republic of China, medical specialty societies, charities, and others: those which only listed author information), guideline classification, classification of diseases, theme (prevention, diagnosis, treatment, prevention and treatment, diagnosis and treatment, nursing, rehabilitation, technical operation, health policy, and prevention and control of infectious diseases), CPGs users, target population, pages of CPGs document, and number of references. The guideline classification was based on the WHO guideline classification method. The diseases were classified according to the International Classification of Disease revision 11 (ICD-11).

The following data were extracted to demonstrate the methodological characteristics: multidisciplinary development teams, systematic literature searching, quality evaluation of included literature, recommendations based on the evidence of systematic reviews, clear criteria of grading the quality of evidence and strength of recommendations used, the factors (such as feasibility, economy, security, equity, acceptability, values, and patient preferences) being considered in the formulation of each recommendation, clear, precise, and actionable recommendations, the level of evidence designated, the strength of recommendations presented, and the conflicts of interest declared.

The definitions used related to the methodological characteristics are stated below. A multidisciplinary development team was described as a diverse group that included more than two kinds of the following representatives: relevant technical experts or health professionals, end-users, representatives of groups most affected by the recommendations, methodologists (assessing evidence and developing guidelines informed by evidence, or health economist or technical experts of equity and human rights). The systematic literature searching meant accessing and searching at least four databases in English and Chinese (e.g., PubMed, Cochrane library, and CNKI). We defined recommendations based on evidence of the systematic reviews of the scientific literature as “at least one piece of the evidence supporting a recommendation came from a systematic review or meta-analysis.” A systematic review was described as “a review of a clearly formulated question that uses systematic and explicit methods to identify, select, and critically appraise relevant research, and to extract and analyze data from the studies that are included in the review.”

### Statistical Analysis

The characteristics of the included guidelines were recorded by two independent reviewers into an EXCEL file. Inter-rater reliability was assessed by the Kappa statistics. The characteristics were summarized and stratified by the year of guideline development. Frequency and percentage were presented for the categorical characteristics. The included guidelines were classified into CB-CPGs and EB-CPGs based on the category or were dichotomized based on whether GRADE was used. Between-group comparisons of the methodological characteristics were conducted using the chi-square test or Fisher's exact test. Mann–Kendall Trend Test (M-K test), a non-Parametric method, was adopted to identify the monotonically increasing or decreasing trends of methodological characteristics over years, a positive *z* value indicated a monotonic upward trend, and a negative one indicated a downward trend. The data were analyzed using SPSS version 22.0 (IBM, NY, USA), and a two-sided *p*-value of < 0.05 was considered as statistically significant.

## Results

### Flow of Included Studies

**Figure 1:**
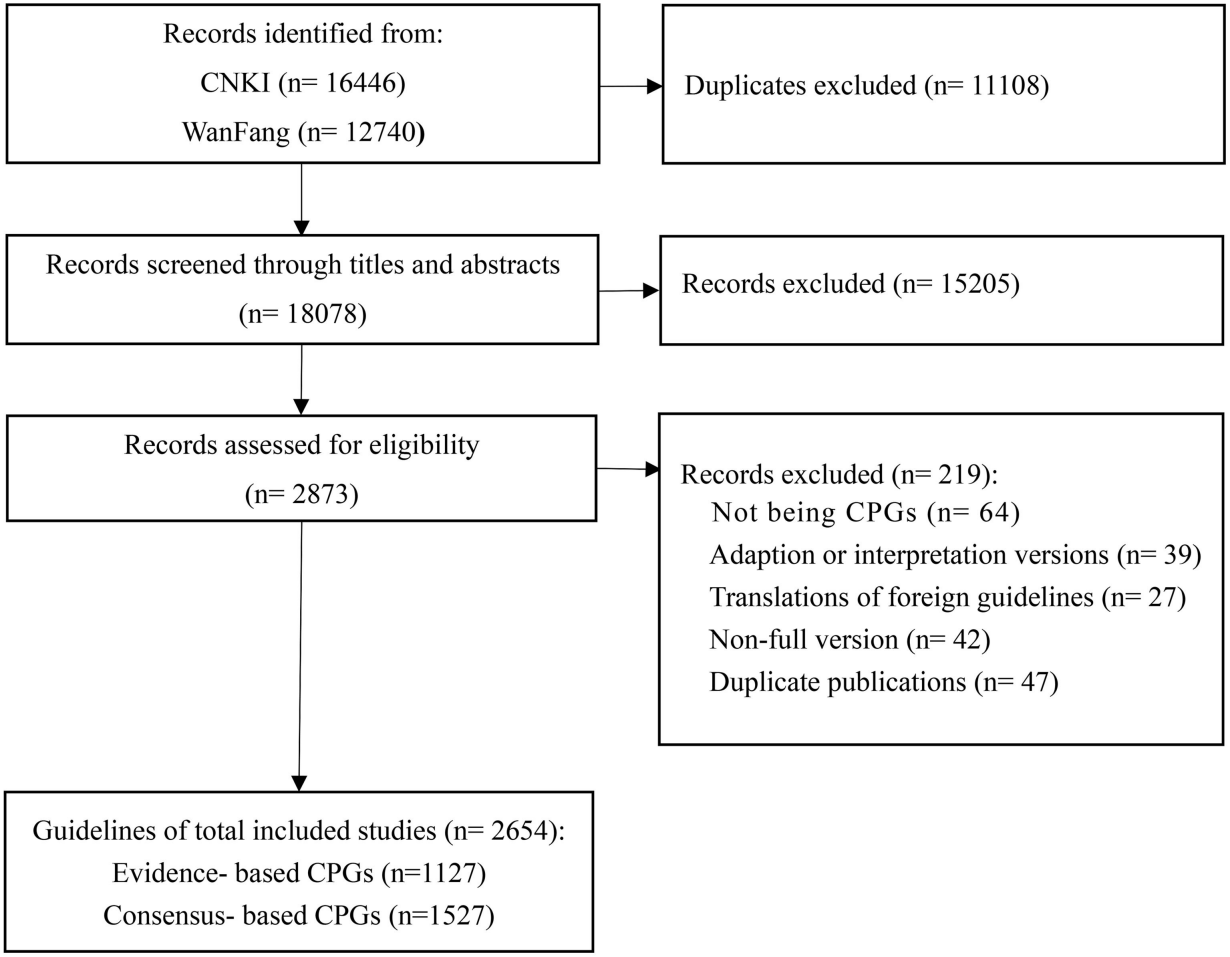
The flowchart for guideline selection.

A total of 29,186 articles were identified, of which 18,078 were considered potentially relevant; after selection, a total of 2,873 guidelines were eligible. In total 2,654 guidelines (EB-CPGs = 1,127, CB-CPGs = 1,527) were selected according to the selection criteria **(**as shown in Figure 1).

### General Characteristics of Guidelines

#### Number and Themes of CPGs

**Figure 2:**
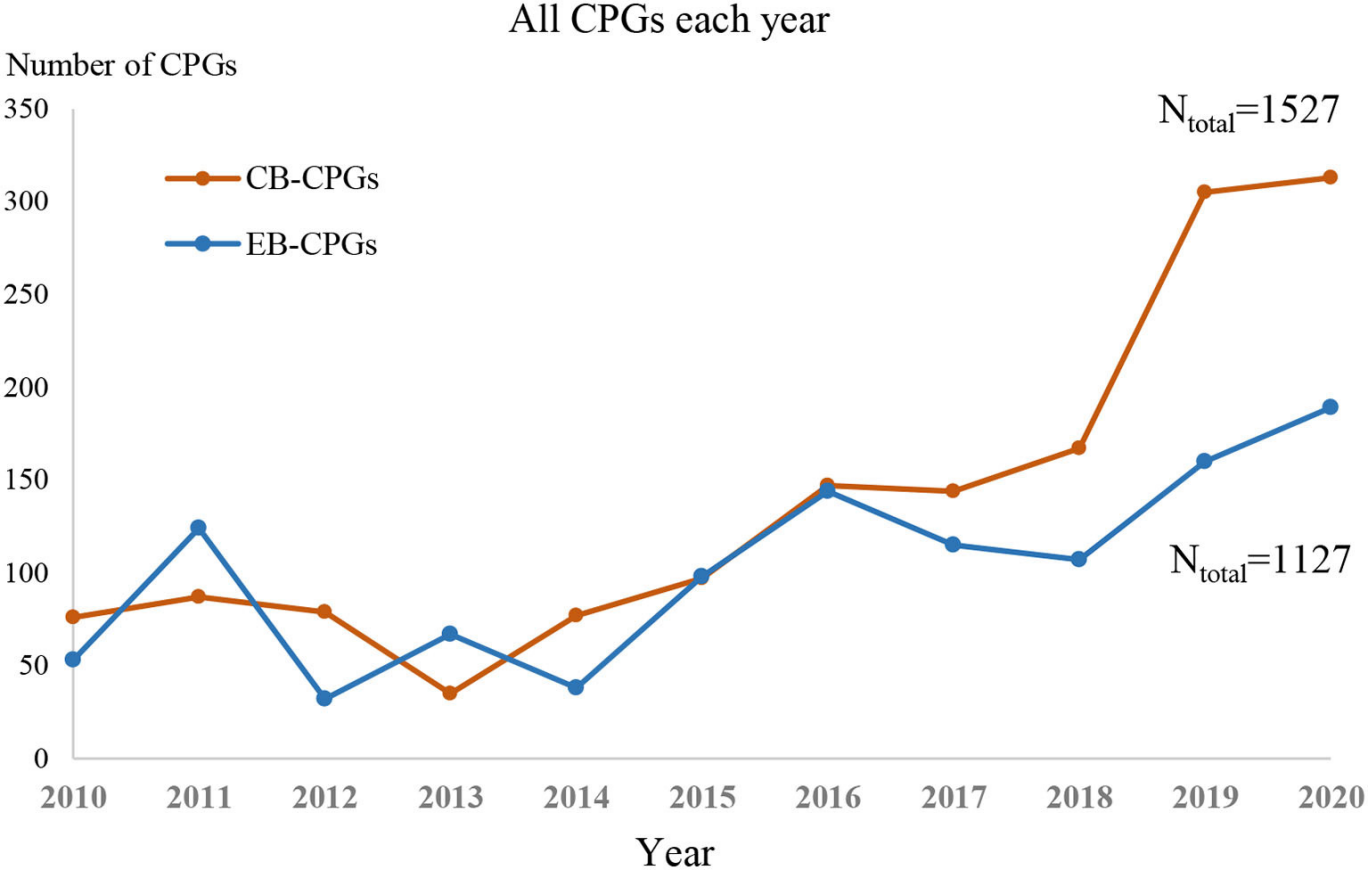
The publication trends of the guidelines from 2010 to 2020.

Between 2010 and 2020, the production of guidelines was increasing annually (as shown in Figure 2). The number of CB-CPGs published in the last 3 years was far more than that of EB-CPGs.

According to the categories of areas covered by the guidelines, 2,318 (87.34%) guidelines focused on Western Medicine, 195 (7.35%) on Traditional Chinese Medicine (TCM), and 141 (5.31%) on the combination of Western Medicine and TCM. Eight (0.30%) patient versions of guidelines were developed. According to the WHO guideline classification method, 2,628 (99.02%) were standard guidelines, 10 (0.38%) were tentative guidelines, and 16 (0.60%) were rapid advice guidelines [including, four guidelines addressing coronavirus disease 2019 (COVID-19)].

**Figure 3:**
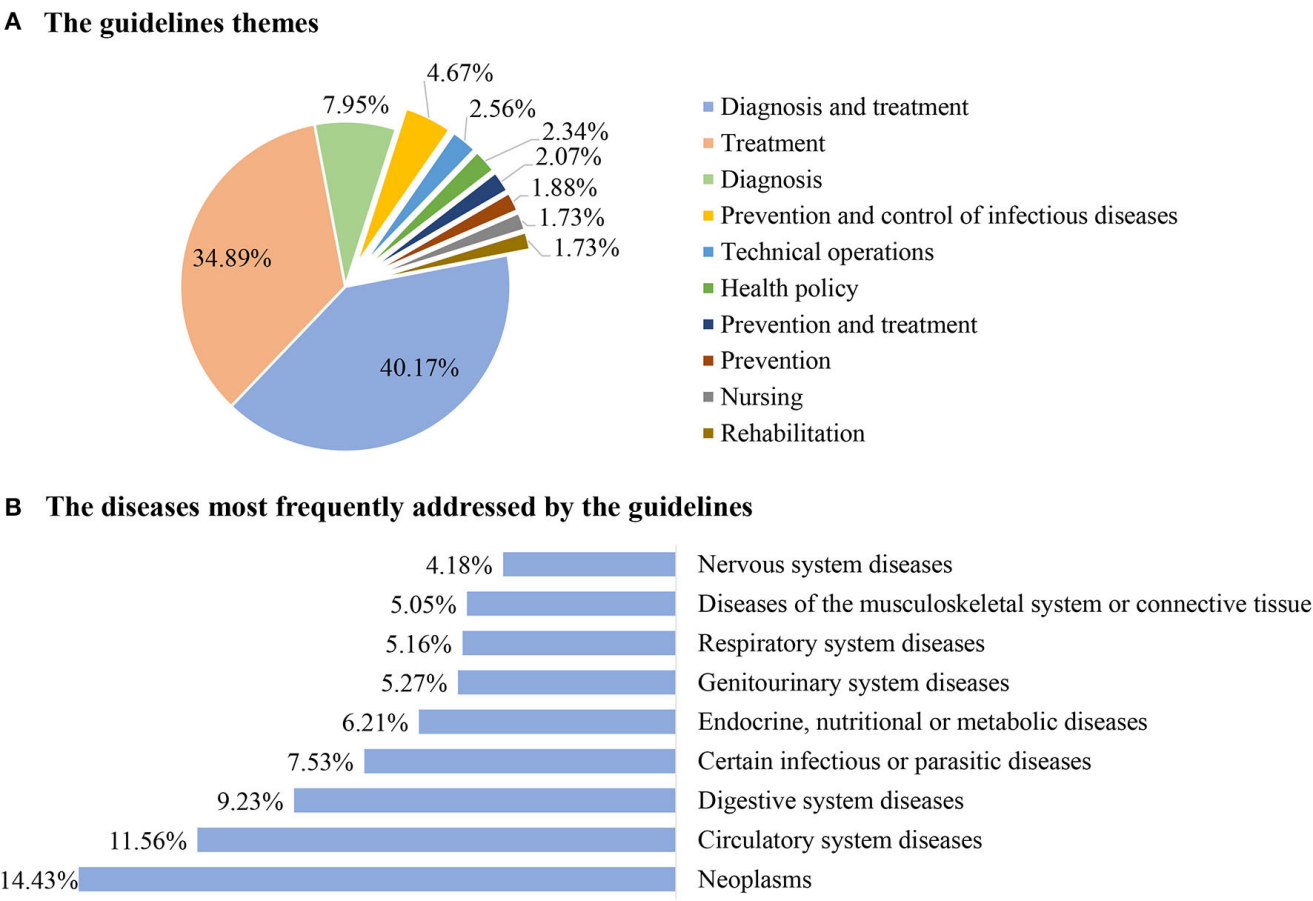
General characteristics of the guidelines from 2010 to 2020. **(A)** The guidelines themes. **(B)** The diseases most frequently addressed by the guidelines.

In the past 11 years,1,066 (40.17%) guidelines have focused on the diagnosis and treatment, 926 (34.89%) on treatment, 211 (7.95%) on diagnosis, 124 (4.67%) on the prevention and control of infectious diseases, 68 (2.56%) on the technical operations, 62 (2.34%) on health policy, 55 (2.07%) on prevention and treatment, 50 (1.88%) on prevention, 46 (1.73%) on nursing, and 46 (1.73%) on rehabilitation (as shown in Figure 3A). Some guidelines were republished between 1 and 20 times, in most cases, this was 1–3 times.

#### Development Organizations and Diseases Addressed by CPGs

The guideline developers included government agencies (the National Health Commission of the People's Republic of China) [69 (2.6%)], the medical specialty societies (including their branches) [1887 (71.1%)], the medical institutions or charities [88 (3.32%)], and other [610 (22.98%)]. The guidelines covered a broad range of diseases. The most addressed diseases were neoplasms (14.43%), followed by the circulatory system diseases (11.56%), digestive system diseases (9.23%), certain infectious or parasitic diseases (7.53%), endocrine, nutritional, or metabolic diseases (6.21%), genitourinary system diseases (5.27%), respiratory system diseases (5.16%), diseases of the musculoskeletal system or connective tissue (5.05%), and nervous system diseases (4.18%) (as shown in Figure 3B).

#### Target Population

In total 75 (2.83%) guidelines addressed the obstetric and gynecological patients, and 324 (12.21%) focused on newborns, infants, and children.

### Methodological Characteristics of Guidelines

**Figure 4:**
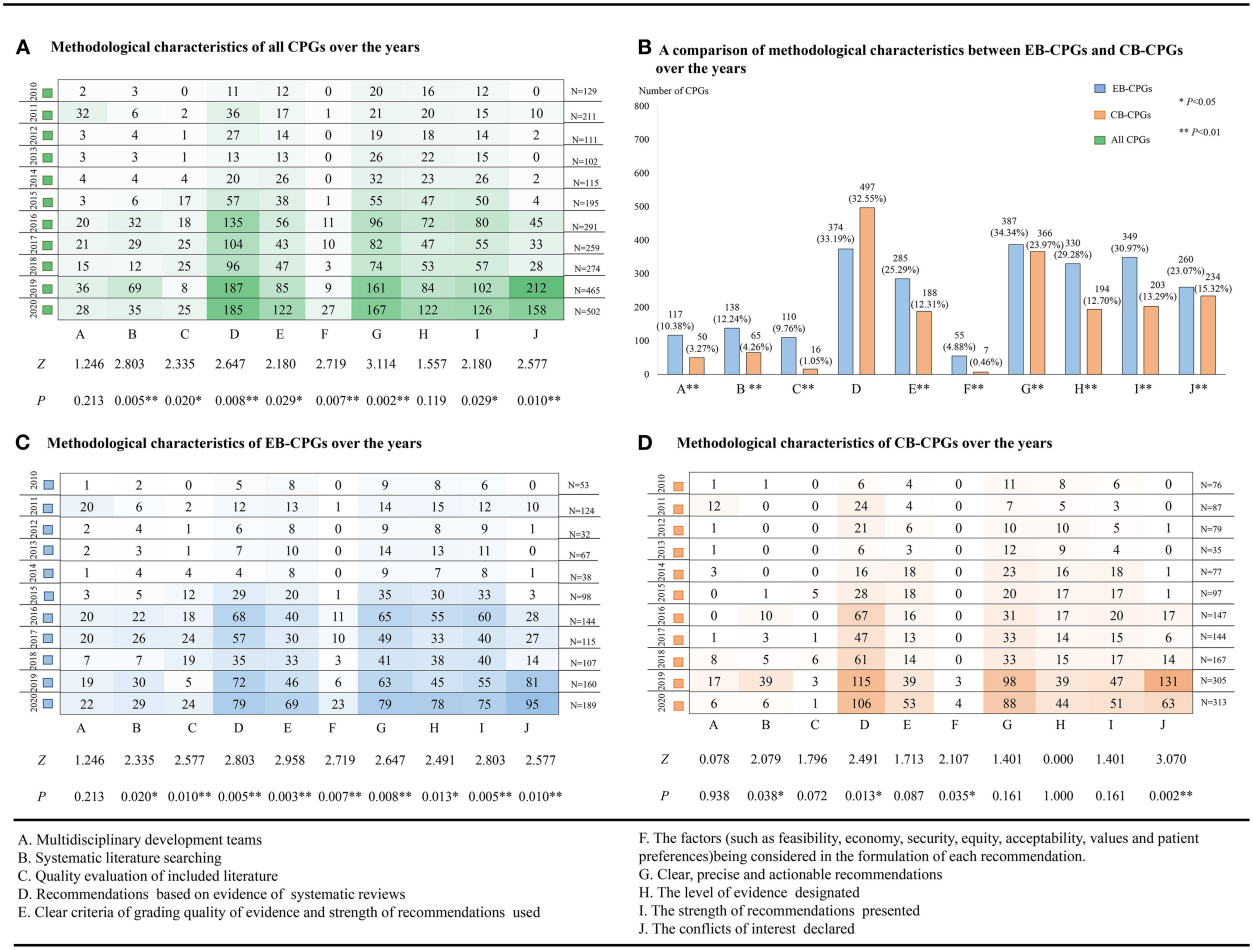
The methodological characteristics of the guidelines from 2010 to 2020. **(A)** The methodological characteristics of all the clinical practice guidelines (CPGs) over the years. **(B)** A comparison of the methodological characteristics between evidence-based (EB)-CPGs and consensus-based (CB)-CPGs over the years. **(C)** The methodological characteristics of EB-CPGs over the years. **(D)** The methodological characteristics of CB-CPGs over the years.

Although the methodological quality of CB-CPGs was clearly lower than the EB-CPGs and affected the total overall quality level of the CPGs, the methodological quality of all CPGs has continued to improve steadily over time (as shown in Figure 4A).

#### Multidisciplinary Development Teams of the Guideline

The majority of the guideline developers included relevant health professionals (e.g., clinicians or nurses) or technical experts, and 167 (6.29%) of guideline groups included at least one methodologist (EBM, statisticians, information retrieval, epidemiologist, health economist, or experts for equity and human rights). The EB-CPGs were significantly higher for this item because the development of 10.38% (117/1,127) EB-CPGs and 3.27% (50/1,527) CB-CPGs were supported by the multidisciplinary development teams (Item A, *P* < 0.01) (as shown in Figure 4B). This methodological quality issue did not improve over the time span in either EB-CPGs (as shown in Figure 4C) or CB-CPGs (as shown in Figure 4D) (Item A, *P* > 0.05). Of all the included guidelines, only three CPGs reported target population (patient) involvement in the guideline development group.

#### Searching and Evaluation of Evidence

Of all 2,654 guidelines, only 7.65% (203/2,654) were based on a full literature search which included 12.24% EB-CPGs (138/1,127) and 4.26% CB-CPGs (65/1,527). It was clear that there was a significant difference between the EB-CPGs and CB-CPGs (Item B, *P* < 0.01) (as shown in Figure 4B). Both the EB-CPGs and CB-CPGs have improved in this area over the time span (Item B, *P* < 0.05) (as shown in Figures 4C,D). In addition, 7.54% (200/2,654) of the guidelines had more than 100 references which included 12.16% (137/1,127) EB-CPGs and 4.13% (63/1,527) CB-CPGs, whereas 298 (11.23%) of the guidelines did not contain any references.

A total of 2,654 guidelines were published, with few guidelines evaluating the quality of the included literature during development which included 110 (9.76%) EB-CPGs and 16 (1.05%) CB-CPGs. Figure 4B describes the significant difference between the EB-CPGs and CB-CPGs (Item C, *P* < 0.01). In addition, Figure 4C shows the improvement in the quality of the EB-CPGs over the time span in this item (Item C, *P* < 0.01).

From 2010 to 2020, there were 32.82% (871/2,654) of the guidelines whose recommendations were based on the evidence of systematic reviews, which consisted of 33.19% (374/1,127) EB-CPGs and 32.55% (497/1,527) CB-CPGs. No significant statistical difference was observed (Item D, *P* > 0.05) (as shown in Figure 4B). However, there was still a significant improvement in the quality of these CPGs over the last 5–6 years. (Item D, *P* < 0.05) (as shown in Figures 4C,D).

#### Grading Quality of Evidence and Strength of Recommendations

In the past 11 years, the criteria for assessing the quality of evidence and grading recommendations were varied. In total, 25.29% (285/1127) EB-CPGs and 12.31% (188/1527) CB-CPGs designated clear criteria for grading the quality of evidence and strength of the recommendations. The EB-CPGs were better developed than the CB-CPGs, and there was a significant statistical difference between the two (Item E, *P* < 0.01) (as shown in Figures 4B–D). Up to 30 criteria have been used in the published guidelines. Table 1 shows the details. Some criteria did not cite sources or references, which were based on the adaptions of the tool to the self-defined criteria of authors to assess the quality of evidence and grading recommendations. The Guidelines using such criteria accounted for 7.87% (209/2,654) of the total number of publications.

**Table 1:** Criteria used in the published guidelines from 2010 to 2020.

**Criteria of grading quality of evidence and strength of recommendations**	**The level of evidence**	**The strength of recommendations**	**The number of EB-CPGs (*n*/%)**	**The number of CB-CPGs (*n*/%)**
The Joanna Briggs Institute Levels of Evidence and Grades of Recommendation (2014)	1, 2, 3, 4, 5	A, B	1/0.35%	–
The criterion of “S3-guidelines” from German Association of Scientific Medicine	Being based on good clinical practice, GCP	A, B, 0	1/0.35%	–
American Academy of Orthopedic Surgeons, AAOS Osteoarthritis Research Society International, OARSl	I, II, III, IV	1, 2, 3	3/1.05%	–
Delphi classification standard proposed by International Infection Forum (ISF) in 2001	I, II, III, IV, V	A, B, C, D, E	17/5.96%	–
Oxford Centre For Evidence Based Medicine, OCEBM (2011)	1a, 1b, 1c, 2a, 2b, 2c, 3a, 3b, 4, 5	A, B, C, D	16/5.61%	12/6.38%
Oxford Centre For Evidence Based Medicine, OCEBM (2001) The Grading of Recommendations, Assessment, Development, and Evaluation, GRADE	I, II, III, IV, V	Strong, Weak	2/0.70%	–
American College of Chest Physicians) Health and Science Policy Grading System, ACCP	Excellent, Good, Bad, Expert opinion	A, B, C, D, I, E/A, E/B, E/C, E/D	2/0.70%	–
The Grading of Recommendations, Assessment, Development, and Evaluation, GRADE	High, Moderate, Low, Very low	Strong, Weak	115/40.35%	72/38.30%
The North American Spine Society, NASS	1, 2, 3, 4, 5	A, B, C, I	4/1.40%	–
Canadian Task Force on Preventive Health Care, CTFPH	I, II-1, II-2, II-3, III	A, B, C, D, E, I	2/0.70%	1/0.53%
US Preventive Services Task Force, PSTF	I, IIa, IIb, IIc, III	A, B, C	1/0.35%	–
Jianping Liu, Evidence Classification of Clinical Research Based on Evidence Body	Ia, Ib, IIa, IIb, IIIa, IIIb, IV, V	Recommended, Selectively Recommended, Not Recommended, Prohibited	10/3.51%	–
American Association for the Study of Liver Diseases, AASLD	A, B, C	I, IIa, IIb, III	3/1.05%	–
American Academy of Neurology, AAN	I, II, III, IV	A, B, C, U	2/0.70%	1/0.53%
American Association of Neurological Surgeons, AANS	I, II, III	1, 2, 3	1/0.35%	–
American Diabetes Association, ADA	A, B, C, D, E	–	1/0.35%	–
Infectious Diseases Society of America, IDSA American Thoracic Society, ATS	I, II, III	A, B, C	1/0.35%	–
National Clinical Guidelines Database Evidence Rating System	Ia, Ib, IIa, IIb, III, IV	A, B, C	3/1.05%	–
American Heart Association, AHA American College of Cardiology, ACC American Stroke Association, ASA European Society of Cardiology, ESC	A, B, C	I, IIa, IIb, III	26/9.12%	10/5.32%
U.S. Preventive Services Task Force, PSTF	Good, Fair, Poor	A, B, C, D, E, I	3/1.05%	1/0.53%
European Federation of Neurological Societies, EFNS	I, II, III, IV	A, B, C, D	1/0.35%	–
Global Asthma Initiative (GINA) standards	A, B, C, D	–	1/0.35%	–
Shouchuan Wang, the grading standards for TCM literature	I, II, III, IV, V	A, B, C, D, E	19/6.67%	4/2.13%
Jiyao Wang, the Evaluation System of Chinese CPGs	A, B, C	Strong, Weak	1/0.35%	–
Royal College of Obstetricians and Gynecologists, RCOG International Society of Ultrasound in Obstetrics and Gynecology, ISUOG	1+, 2++, 2+, 2-, 3	A, B, C, D	8/2.81%	–
American College of Obstetricians and Gynecologists, ACOG	I, II-1, II-2, II-3, III	A, B, C, D, E, I	1/0.35%	–
Consensus of cerebrovascular group of Chinese Neurology Association	A, B, C, D	I, II, III, IV	10/3.51%	–
The standard of Global Initiative for Prevention and Treatment of Bronchial Asthma (GINA)	A, B, C, D	–	–	1/0.53%
International Endoscopic Hernia Society	1A, 1B, 2A, 2B, 2C, 3, 4, 5	A, B, C, D	–	1/0.53%
Micromedex's Thomson grading system	A, B, C	I, IIa, IIb, III	–	1/0.53%
UK Cochrane Centre for Evidence Classification U.S Preventive Services Task Force, PSTF	1a, 1b, 1c, 2a, 2b, 3a, 3b, 4, 5	A, B, C, D, I	–	2/1.06%
revised Scottish Intercollegiate guide—lines network (SIGN)grading system	1++, 1+, 1L, 2++, 2+, 2L, 3, 4	A, B, C, D	–	1/0.53%
National Comprehensive Cancer Network, NCCN	1, 2A, 2B, 3	–	–	1/0.53%

**Figure 5:**
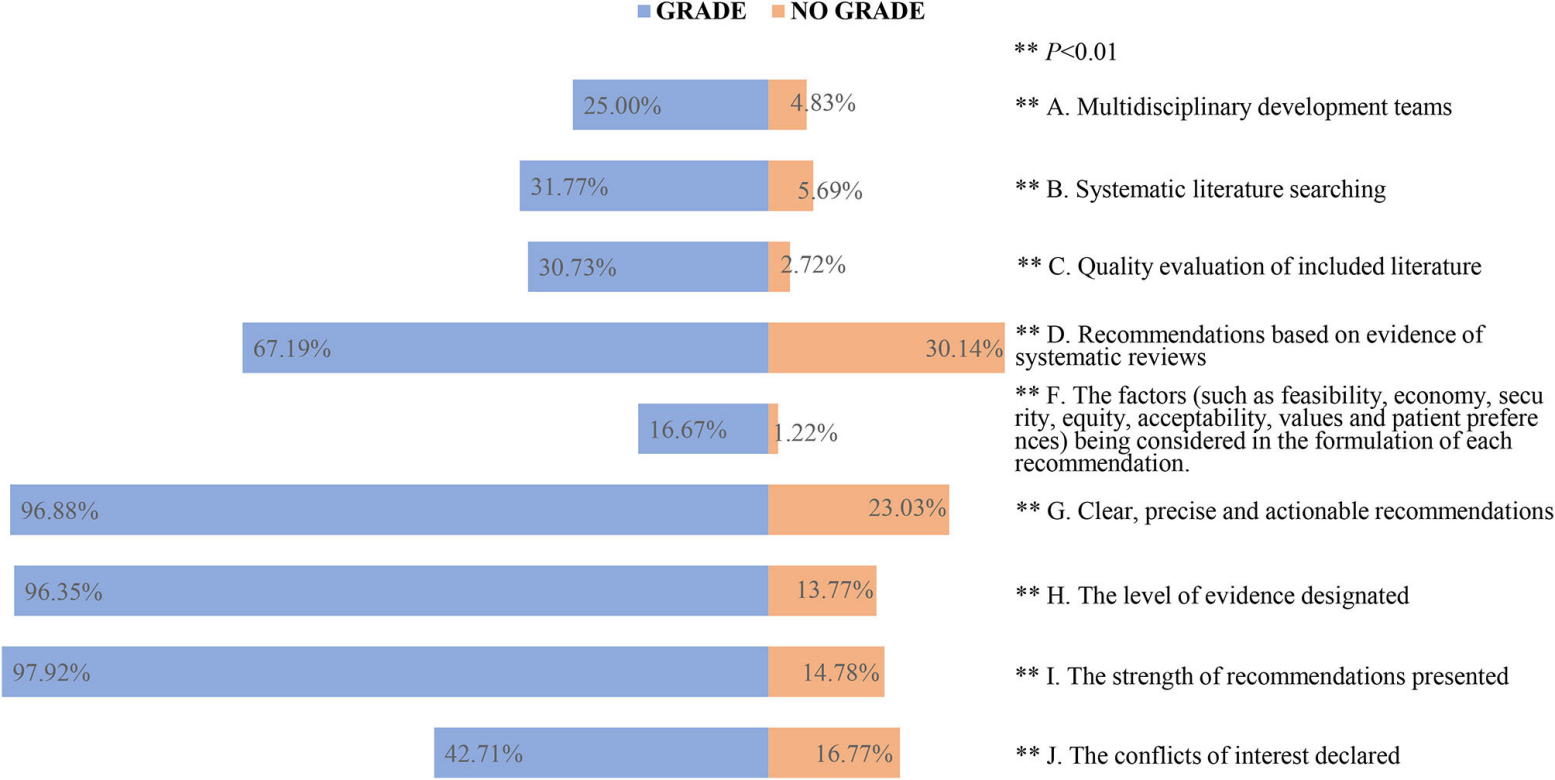
The methodological characteristics in the guidelines using and not using GRADE.

For the past 11 years, there were 7.23% (192/2,654) guidelines that used GRADE methodology of which 10.65% (120/1,127) were EB-CPGs and 4.72% (72/1,527) were CB-CPGs. We analyzed information about the methodological characteristics and compared those using GRADE with those not using it to assess the certainty of the evidence and/or formulate the recommendation. Among the guidelines which used GRADE vs. those that did not, we observed higher methodological quality in the guidelines using GRADE (*P* < 0.01). Figure 5 shows the details.

#### Developing Recommendations

In total, 753 (28.37%) guidelines described clear, precise, and actionable recommendations, but only 62 (2.34%) considered the factors, such as feasibility, economy, equity, acceptability, values, and preferences of the patient in the formulation of each recommendation of which 48 guidelines considered values and preferences of the patient, 38 guidelines considered economic issues, and two considered equity issues. No GRADE evidence to decision (ETD) tool or other tools were used in the process of developing recommendations. There was a statistically significant difference between 4.88% (55/1127) EB-CPGs and CB-CPGs 0.46% (7/1527) (Item F, *P* < 0.01), which is shown in Figure 4B. Both the EB-CPGs and CB-CPGs have improved in this methodological characteristic over the time span (Item F, *P* < 0.05) (as shown in Figures 4C,D).

#### Presentation of the Recommendations

A total of 387 (34.34%) EB-CPGs described clear, precise, and actionable recommendations, among which 330 (29.28%) designated the level of evidence, 349 of 387 (30.97%) presented the strength of recommendations, and 300 of 387 (26.62%) presented both the level of evidence and the strength of recommendations. In addition, 366 (23.97%) CB-CPGs present clear, precise, and actionable recommendations, among which 194 (12.70%) have designated the level of evidence, 203 of 366 (13.29%) presented the strength of recommendations, and 161 of 366 (10.54%) presented both the level of evidence and the strength of recommendations. Compared with the CB-CPGs, the EB-CPGs have improved significantly in this item over the time span (Item G, H, and I, *P* < 0.01) (as shown in Figures 4C,D). The significant differences between EB-CPGs and CB-CPGs were observed in this methodological characteristic (Item G, H, and I, *P* < 0.01) as shown in Figure 4B.

#### Sources of Funding for the Guidelines and Conflicts of Interest

In total, 19.89% (528/2,654) of the guidelines were funded during the development; the EB-CPGs were 22.8% (257/1,127) and the CB-CPGs were17.75% (271/1,527). Most of the funding (59.28%, 313/528) came from the national government departments, such as 12.18% (96/528) The National Natural Science Foundation of China and 10.04% from the Ministry of Science and Technology of the People's Republic of China. In addition, 26.85% (69/257) were supported by the National Administration of Traditional Chinese Medicine. Furthermore, 0.72% (19/2654) guidelines reported that they received funding from the companies and four guidelines stated that the companies did not participate in or influence the academic content of the guidelines or the evaluation of the evidence.

Indeed, 18.61% (494/2,654) of the guidelines declared the result of identifying and managing conflicts of interest, which included 23.07% (260/1,127) EB-CPGs and 15.32% (234/1,527) CB-CPGs. Although all the CPGs have improved in this area of the methodological quality over the time span, a significant statistical difference still remains between the EB-CPGs and CB-CPGs (Item J, *P* < 0.01) (as shown in Figures 4B–D). Of all the CPGs, only 1.36% (36/2,654) reported on the collection and review of methodology for conflict of interest. None of the guidelines provided publicly accessible conflict disclosure statements for all the authors.

## Update

In total, 11.27%(127/1,127)EB-CPGs and 8.38% (128/1,527) CB-CPGs have been updated. These updated guidelines ranged from 1 to 4 times, with 203 (7.65%) updated one time, 35 (1.32%) updated two times, 14 (0.53%) updated 3 times, and 3 (0.11%) updated 4 times. The longest update interval was 10 years, the shortest was 1 year, and 150 (58.82%) guidelines were updated within 2–4 years.

## Discussion

In this study, a large number of descriptive and analytic data were used to analyze the general characteristic information and the methodological characteristic of guidelines over the past 11 years in China. The results showed that the quantity and quality of the guidelines developed in China have improved over the time span. Compared with a previous study evaluating the guidelines from 1993 to 2010 (3), significantly better guideline quality was observed in the 2010–2020 time period, even in the CB-CPGs. Most of the guidelines were developed by the medical specialty societies (including their branches), and the government agencies could not be completely separated from them in developing the CPGs. More than a quarter of guidelines described clear, precise, and actionable recommendations. More than 10% focused on the TCM or combination of Western Medicine and TCM. In addition, we saw patient versions of guidelines and rapid advice guidelines which were developed in recent years.

But the challenge is still conspicuous. <10% of guidelines included methodologists in the development group and patient involvement was seldom evident. We have included 1,127 EB-CPGs, as they do not fulfill the evidence-based guidelines criteria which include reporting a search strategy, classifying the quality of evidence, and grading the strength of recommendations. Only slightly more than 10% of EB-CPGs were based on the full literature search, and about a quarter had used clear criteria for grading the quality of evidence and strength of the recommendations. In addition, we found that a significant portion of CPGs did not declare information about the conflict of interest, and all lacked the conflict of interest management, which was quite different from the research data reported abroad (14, 15). The conflict of interest management in the clinical practice guidelines will always be a focus (16). The methodological quality of CB-CPGs was obviously lower than the EB-CPGs. Although a previous study indicated that expert consensus in China was usually developed without any formal approach, did not use evidence symmetrically, and seldom dealt with the conflicts of interests (7), in our study, some CB-CPGs paid attention to the methodology quality requirements.

The usage rate of GRADE has improved in recent years but is still low. The guidelines using GRADE had a higher methodological quality which is consistent with guideline assessment in the recent publications (17–19). In addition, the data showed that the timely updated guidelines, multidisciplinary development teams, transparent management of COI, explicit link between the supporting evidence and recommendation remained low especially in the CB-CPGs.

The guidelines we included were described in Chinese as “clinical practice guideline” or “expert consensus” in Chinese guideline documents which may not be a reasonable description. Given that all the guidelines have a consensus process, Djulbegovic and Guyatt have suggested classifying all the CPGs into two categories: EB-CPGs and non-EB-CPGs, so as to avoid using the term “CB-CPGs” (20). In this study, we have designated “CBG” or “expert consensus” as EB-CPGs and CB-CPGs as nearly approximating to the Chinese word guideline developers used in the title and nearly all the CB-CPGs conducted a search and referred evidence at different levels. We think that there is no time when no evidence existed even in the early stages of a newly identified infection. For example, early in 2020, the researchers published a rapid advice guideline for the diagnosis and treatment of COVID-19 based on the indirect evidence (21), such as that for severe acute respiratory syndrome (SARS), Middle East respiratory syndrome (MERS), and expert evidence through a structured form to collect this information from case reports, summaries, and reports from first-line clinicians.

The methodological quality of the EB-CPGs was significantly better than that of the CB-CPGs possibly because of loose methodological requirements of the latter's formulation leading to a simpler and faster development process. Unfortunately, the formulation of CB-CPGs, which ignores the process of synthesizing the best evidence, will be bound to give different and potentially erroneous advice (22). It is a misunderstanding that the evidence-based guidelines can be only developed if well-designed controlled trials exist. The EBM principles apply equally well to low- or high-quality evidence and the situations when only low-quality evidence is available may be those in which clinicians most need guidance (20). Overall, we hypothesize that at any time, the guidelines should be evidence based and where the best evidence is not available, then the best available evidence needs to be used.

In China, most guidelines were developed by the government agencies and the medical specialty societies (including their branches), which was consistent with a recently published study (23). Therefore, they should take responsibility for ensuring that the guidelines are normative and scientific. They should have a complete guideline development and evaluation system, with the methodological and reporting standards. Multidisciplinary participation, especially of methodologists, should be encouraged in the guideline development. Then, the Editors should strictly review the quality of guidelines before they are published. This study clearly shows that the conflict of interest management in the Chinese guidelines is still in its infancy and the awareness of developers is quite weak. Conflicts of interest must be properly managed during the development of the guidelines. Systematic literature searching should be conducted at all times in the guideline development process, and the reasons for using expert consensus need to be justified. The development of EB-CPGs is the trend of the future, not CB-CPGs.

To ensure the complete exploration of the status and trends of Chinese CPGs, we included EB-CPGs and CB-CPGs including all levels of developers or organizations no matter which themes the guideline focused on. This systematic review of the status of Chinese CPGs has covered a wide range of subjects, such as the general characteristics and the methodological characteristics over the past decade in China. To our knowledge, this is the first study to examine a large number of CB-CPGs by exploring their methodological quality, and it gives scientific conclusions by comparing the detailed data with EB-CPGs. Here are some suggestions for improving the quality of guidelines: (a) multidisciplinary participation, especially of methodologists, should be encouraged in guideline development and systematic literature searching should be conducted at all times in the guideline development process; (b) clear criteria, such as GRADE, for assessing the quality of evidence and grading recommendations should be applied; (c) the guideline developers especially the medical specialty societies (including their branches) should adhere to the accepted methodological and reporting standards in the guideline development; (d) conflicts of interest must be properly managed during the development of the guidelines; (e) the Editors should strictly review the quality of guidelines before they are published; and (f) the guidelines should at all times be evidence based, and consensus should just be used as a process for forming the recommendations.

The systematic review has certain limitations. First, we did not use the AGREE II instrument to assess the quality of the included guidelines. Although we extracted and compared many methodological characteristics in which we were interested between the EB-CPGs and CB-CPGs, they may not comprehensively reflect the quality of the included guidelines. Second, we did not search websites of the National Health Commission or the medical specialty societies maybe leading to publication bias. Third, we defined recommendations based on a systematic review of the scientific literature as “at least one of evidence supported recommendations came from systematic review or meta-analysis.” During data extraction, we noticed that the evidence references for developing recommendations comprised a significantly different proportion of different guidelines, recommendations; with some guidelines citing inappropriate or insufficient evidence although they had cited a systematic review.

## Conclusion

This study shows that the quantity and quality of the guidelines developed in China have been improved over the time span of our study. But this is patchy across different methodological quality criteria. Although there are some obstacles to good practice that are needed to overcome in the development of guidelines, the CPGs will continue to play a critical role in helping the decision-making in the medical environment.

## Data Availability Statement

The raw data supporting the conclusions of this article will be made available by the authors, without undue reservation.

## Author Contributions

Y-hJ and Y-yW designed the study and formulated inclusion criteria. S-yY, X-yR, and Y-yW searched and identified eligible guidelines. RZ and Y-yW extracted important information. RZ, QH, RT, Z-rZ, and Y-hJ analyzed the data. RZ, L-xS, Y-bW, Y-qD, and Y-hJ contributed to the discussion of the findings. RZ, X-yR, and Y-hJ developed the final manuscript. All the authors have read and approved the manuscript.

## Funding

This work was supported (in part) by the National Natural Science Foundation of China (Grant No. 82174230) and Translational Medicine and Interdisciplinary Research Joint Fund of Zhongnan Hospital of Wuhan University (Grant No. ZNJC202016). The research funds are mainly used to provide the labor expenses of researchers and the expenses for the publication of articles.

## Conflict of Interest

The authors declare that the research was conducted in the absence of any commercial or financial relationships that could be construed as a potential conflict of interest.

## Publisher's Note

All claims expressed in this article are solely those of the authors and do not necessarily represent those of their affiliated organizations, or those of the publisher, the editors and the reviewers. Any product that may be evaluated in this article, or claim that may be made by its manufacturer, is not guaranteed or endorsed by the publisher.
